# Supplementary material for: Auxology of small samples: A method to describe child growth when restrictions prevent surveys
Source: PLoS One. 2022 Jun 7;17(6):e0269420. doi: 10.1371/journal.pone.0269420 (PMC9173602; doi:10.1371/journal.pone.0269420)
Supplement: Attachment — Submitted filename: renamed_32d1c.docx [file pone.0269420.s002.docx]

Replies to the Editor and reviewers

Journal Requirements:

1. When submitting your revision, we need you to address these additional requirements.

Please ensure that your manuscript meets PLOS ONE's style requirements, including those for file naming. The PLOS ONE style templates can be found at 
https://journals.plos.org/plosone/s/file?id=wjVg/PLOSOne_formatting_sample_main_body.pdf and 
<https://journals.plos.org/plosone/s/file?id=ba62/PLOSOne_formatting_sample_title_authors_affiliations.pdf>

We have followed style requirements

2. Please provide additional details regarding participant consent. In the ethics statement in the Methods and online submission information, please ensure that you have specified what type you obtained (for instance, written or verbal, and if verbal, how it was documented and witnessed). If your study included minors, state whether you obtained consent from parents or guardians. If the need for consent was waived by the ethics committee, please include this information.

We have specified types of consent for both South African and Polish children in the Methods section giving names of ethics committees and describing how individual consent, and consent of parents/guardians was obtained. See lines 90-104 and 117-122 of the revised manuscript.

3. Our policy for research in this area aims to improve transparency in the reporting of research performed outside of researchers’ own country or community. The policy applies to researchers who have travelled to a different country to conduct research, research with Indigenous populations or their lands, and research on cultural artefacts. The questionnaire can also be requested at the journal’s discretion for any other submissions, even if these conditions are not met.  Please find more information on the policy and a link to download a blank copy of the questionnaire here: https://journals.plos.org/plosone/s/best-practices-in-research-reporting. Please upload a completed version of your questionnaire as Supporting Information when you resubmit your manuscript.

Both in South Africa and in Poland research was conducted in researchers’ own countries in local communities with whom researchers had personal ongoing contacts. Filled out Questionnaire is submitted.

4. We noted in your submission details that a portion of your manuscript may have been presented or published elsewhere. 
(PLOS Medicine)
Please clarify whether this [conference proceeding or publication] was peer-reviewed and formally published. If this work was previously peer-reviewed and published, in the cover letter please provide the reason that this work does not constitute dual publication and should be included in the current manuscript.

NO, no portion of our manuscript was published elsewhere as far as we can remember. internet searches failed to discover any such publication. Checked PLOS Medicine – negative result. Our paper is based on data used for other studies that had aims and methods different from the current one. For this reason, materials and some characteristics of studied children have been described in a number of papers, cited in the references to the current paper. None of these papers, however, uses the method, the approach and the results produced for the current paper. No refereed conference proceeding or publication has a content similar to the current paper.

A paper with a similar title, but different contents “Auxology of small samples: new approach applied to children and adolescents in three Aboriginal communities in Australia” has been submitted to PLOS One by Dr Żądzińska in 2015 (PONE-D-15-13511) and rejected. This paper used data sets from completely different communities (Aboriginal Australians, not African and Polish) and attempted to apply a method similar to the one in the current paper, but not identical, to a different type of data (longitudinal, not cross-sectional) with the aim of characterising growth velocities, not simply the distance growth lines, as the current paper does. Introduction to this rejected paper had some sentences similar, but not identical, to those used in the current paper. Six years after the mentioned rejection, we have done a completely new work on different data sets with altered methods, and aims and results different from the rejected paper.

5. We note that you have indicated that data from this study are available upon request. PLOS only allows data to be available upon request if there are legal or ethical restrictions on sharing data publicly. For more information on unacceptable data access restrictions, please see <http://journals.plos.org/plosone/s/data-availability#loc-unacceptable-data-access-restrictions>.

We now are providing a Supplementary file with all data used for the current paper, no indication that data will be available upon request is made.

In your revised cover letter, please address the following prompts:

a) If there are ethical or legal restrictions on sharing a de-identified data set, please explain them in detail (e.g., data contain potentially sensitive information, data are owned by a third-party organization, etc.) and who has imposed them (e.g., an ethics committee). Please also provide contact information for a data access committee, ethics committee, or other institutional body to which data requests may be sent. No need for this indication. Data are now provided in the Supplement

b) If there are no restrictions, please upload the minimal anonymized data set necessary to replicate your study findings as either Supporting Information files or to a stable, public repository and provide us with the relevant URLs, DOIs, or accession numbers. For a list of acceptable repositories, please see <http://journals.plos.org/plosone/s/data-availability#loc-recommended-repositories>.

The anonymised data set has been uploaded as the Supplementary Information

We will update your Data Availability statement on your behalf to reflect the information you provide.

6. Please include your full ethics statement in the ‘Methods’ section of your manuscript file. In your statement, please include the full name of the IRB or ethics committee who approved or waived your study, as well as whether or not you obtained informed written or verbal consent. If consent was waived for your study, please include this information in your statement as well.

Done, see lines 90-105 and 118-123 of the revised manuscript

7. Please include captions for your Supporting Information files at the end of your manuscript, and update any in-text citations to match accordingly. Please see our Supporting Information guidelines for more information: <http://journals.plos.org/plosone/s/supporting-information>.

Done

8. Please review your reference list to ensure that it is complete and correct. If you have cited papers that have been retracted, please include the rationale for doing so in the manuscript text, or remove these references and replace them with relevant current references. Any changes to the reference list should be mentioned in the rebuttal letter that accompanies your revised manuscript. If you need to cite a retracted article, indicate the article’s retracted status in the References list and also include a citation and full reference for the retraction notice.

Refences checked

[Note: HTML markup is below. Please do not edit.]

Reviewers' comments:

Reviewer's Responses to Questions

**Comments to the Author**

1. Is the manuscript technically sound, and do the data support the conclusions?

The manuscript must describe a technically sound piece of scientific research with data that supports the conclusions. Experiments must have been conducted rigorously, with appropriate controls, replication, and sample sizes. The conclusions must be drawn appropriately based on the data presented.

Reviewer #1: Yes

Reviewer #2: Yes

2. Has the statistical analysis been performed appropriately and rigorously?

Reviewer #1: Yes

Reviewer #2: Yes

3. Have the authors made all data underlying the findings in their manuscript fully available?

The [PLOS Data policy](http://www.plosone.org/static/policies.action#sharing) requires authors to make all data underlying the findings described in their manuscript fully available without restriction, with rare exception (please refer to the Data Availability Statement in the manuscript PDF file). The data should be provided as part of the manuscript or its supporting information, or deposited to a public repository. For example, in addition to summary statistics, the data points behind means, medians and variance measures should be available. If there are restrictions on publicly sharing data—e.g. participant privacy or use of data from a third party—those must be specified.

Reviewer #1: Yes

Reviewer #2: No, now the file S1 dataset containing all data used is submitted

4. Is the manuscript presented in an intelligible fashion and written in standard English?

PLOS ONE does not copyedit accepted manuscripts, so the language in submitted articles must be clear, correct, and unambiguous. Any typographical or grammatical errors should be corrected at revision, so please note any specific errors here.

Reviewer #1: Yes

Reviewer #2: Yes

5. Review Comments to the Author

Reviewer #1: The study proposes a unique method which is parsimonious and efficient in relation to previous methods such as parametric methods. Essentially, the non-parametric method developed describes growth of South African and Polish children using various morphometric elements.

The language of the paper is satisfactory and accessible, with the exception of a few grammatical errors. The method is clearly and logical described.

The results produced are based on the sample size are significant. Also, the study utilises continuous growth curves to a small sample size. Groups of 50 children were randomly selected from a very large cohort. The number of children is satisfactory in my opinion as it does not detract from the study’s hypothesis. The results obtained incorporate the application of polynomial curves in order to show auxological features of the groups.

The method is ideal as it considerably cuts field time and measuring large amounts of individuals which can be problematic due to limited fieldwork time, conflict/war, or bureaucratic issues. The study addresses an old but important issue of the correlation between sample size and reliable information. Does more mean better? Or is less a better approach in extrapolating tenable results? The study provides a testable hypothesis which deserves further attention in auxological studies.

Reviewer #2: This is a very good manuscript.

Only 2 points need to be addressed. They are:

1) The limitation of this study must be mentioned.

Limitations were spelt out in lines 214-216 please note that transmission of the manuscript Word file seems to shift line numbers. For this reason we have highlighted by a “comment” appropriate parts of the text

2) The utility of this paper must be highlighted.

The utility has been highlighted in lines  221-226 and 228-232 please note that transmission of the manuscript Word file seems to shift line numbers. For this reason we have highlighted by a “comment” appropriate parts of the text

Since we have already mentioned the limitations and the utility of our study in the text as indicated above, we can only interpret comments of the reviewer as requiring us to put those lines under separate section subtitles. Such practice, in a short paper, would unnecessarily disrupt the flow of our brief “Discussion” that is almost entirely devoted to limitations and utility of our method.

Minor revisions are required. Done, described above

The request of the Academic Editor in consultation with the journal editors, has been satisfied by adding the following text at lines 252-256:

“In the past anthropometric methods were used, incorrectly, to define “ethnic” or “racial” differences between populations. Since human anthropometric variation is continuous and reflects adaptive responses of human bodies to their immediate living conditions in addition to possible heritable differences, the results of this paper should not be used for any attempts to define taxonomic differences among human populations.“

The authors are firmly opposed to distinguishing biological subdivisions of our species. Human variation is predominantly individual and differences among populations do not justify separating them as categories or breeds, races or subspecies. MH lived in South Africa through the end of apartheid and participated in the first free elections there.

6. PLOS authors have the option to publish the peer review history of their article ([what does this mean?](https://journals.plos.org/plosone/s/editorial-and-peer-review-process#loc-peer-review-history)). If published, this will include your full peer review and any attached files.

If you choose “no”, your identity will remain anonymous but your review may still be made public.

**Do you want your identity to be public for this peer review?** For information about this choice, including consent withdrawal, please see our [Privacy Policy](https://www.plos.org/privacy-policy).

Reviewer #1: No

Reviewer #2: **Yes:** Kaushik Bose

While revising your submission, please upload your figure files to the Preflight Analysis and Conversion Engine (PACE) digital diagnostic tool, <https://pacev2.apexcovantage.com/>. PACE helps ensure that figures meet PLOS requirements. To use PACE, you must first register as a user. Registration is free. Then, login and navigate to the UPLOAD tab, where you will find detailed instructions on how to use the tool. If you encounter any issues or have any questions when using PACE, please email PLOS at [figures@plos.org](mailto:figures@plos.org). Please note that Supporting Information files do not need this step.
